# Enzymatic Production of Collagen Oligopeptides from Porcine Skin and Their Structure–Activity Relationships in Anti-Aging and Skin-Whitening Effect

**DOI:** 10.3390/foods15030507

**Published:** 2026-02-01

**Authors:** Ying-Yan Liang, Hua-Bin Jiang, Sun-Qiang Xu, Li Chen, Zhuo-Han Cai, Xia Wang, Gui-Can Bi, Jun Xie

**Affiliations:** 1 Key Laboratory of Energy Plants Resource and Utilization, Ministry of Agriculture and Rural Affairs, Guangdong Engineering Technology Research Center for Agricultural and Forestry Biomass, College of Future Biomass, South China Agricultural University, Guangzhou 510642, China; l1394806974@163.com (Y.-Y.L.); jianghuabin12@163.com (H.-B.J.); xsqfrank@163.com (S.-Q.X.); bjda02jcl@163.com (L.C.); 13825589020@163.com (Z.-H.C.); xiejun@scau.edu.cn (J.X.); 2 School of Biological and Food Processing Engineering, Huanghuai University, Zhumadian 463000, China; 3 School of Health Management and Biotechnology, Guangdong Food and Drug Vocational College, Guangzhou 510642, China

**Keywords:** collagen oligopeptides, enzymatic preparation, structure–activity relationship, peptideomics, circular dichroism spectroscopy

## Abstract

Collagen-derived peptides are widely studied for their potential roles in skin health and anti-aging. This study applied response surface methodology to optimize the enzymatic hydrolysis of porcine skin-derived collagen oligopeptides (PCOPs) and investigate the associations between peptide characteristics and their cellular effects. The optimized hydrolysis conditions were a solid-to-liquid ratio of 1:2, 52.3 °C, 0.9% enzyme dosage, and pH 7.0. The resulting PCOPs contained 85.77% peptides with molecular weight < 1000 daltons (Da) and 9.68% hydroxyproline. In vitro, 5 mg/mL PCOPs reduced hydrogen peroxide (H_2_O_2_)-induced fibroblast senescence by 39.66% and significantly (*p* < 0.05) reduced tyrosinase activity and melanin synthesis in melanoma cells (B16). Peptidomic profiling identified 52 peptides mainly derived from type I collagen, enriched in Pro-Gly motifs. Circular dichroism analysis indicated that PCOPs primarily consisted of β-sheets (35.3%) and random coils (38.9%). These results suggest that low molecular weight, high hydroxyproline content, Pro-Gly-enriched peptides, and the predominance of β-sheet/random coil structures are associated with the observed cellular effects on fibroblast function and melanogenesis.

## 1. Introduction

As global population aging accelerates and public awareness of health and wellness increases, skin health management and anti-aging strategies have attracted growing attention in biomedical and cosmeceutical research. Skin aging is closely associated with disruptions in extracellular matrix (ECM) homeostasis, particularly collagen degradation induced by intrinsic aging and extrinsic stressors such as ultraviolet (UV) irradiation, leading to wrinkle formation, loss of elasticity, and hyperpigmentation [1,2]. Consequently, supplementation with bioavailable collagen components—particularly collagen peptides—through functional foods and topical formulations has attracted increasing research interest as a potential strategy to support skin homeostasis within the framework of nutricosmetics.

Collagen is the predominant structural protein of the mammalian ECM; however, its high molecular weight and rigid supramolecular organization limit oral bioavailability and transdermal permeability [3,4,5]. Collagen extraction and hydrolysis methods are generally classified into chemical, physical, and enzymatic approaches [6,7,8,9,10,11], among which enzymatic hydrolysis has been widely applied to produce low-molecular-weight collagen oligopeptides with improved absorption characteristics. Notably, peptide fractions below 1000 Da have been reported to exhibit enhanced intestinal permeability and favorable in vivo bioavailability [12,13,14]. Previous studies further suggest that low-molecular-weight collagen-derived peptides are associated with antioxidant activity, fibroblast-related cellular responses, and the modulation of biological processes involved in skin aging and melanogenesis [15,16,17,18,19,20]. In addition, hydroxyproline (Hyp)-enriched peptide motifs have been reported to influence fibroblast-associated responses, potentially contributing to collagen synthesis and antioxidant defense, which may be relevant to cellular stress responses and processes associated with cellular senescence [6,21]. Nevertheless, current enzymatic preparation strategies often result in heterogeneous molecular weight distributions, and the relationships between peptide composition, structural features, and biological activities remain insufficiently understood.

Porcine skin, a major byproduct of the meat-processing industry, represents an abundant and sustainable collagen source with favorable biocompatibility and high hydroxyproline content [22,23]. Although several studies have reported specific bioactivities of porcine skin-derived collagen peptides [13,24,25], systematic investigations integrating preparation optimization, molecular characterization, and bioactivity evaluation of porcine skin collagen oligopeptides (PCOPs) remain limited. In particular, the combined influence of molecular weight distribution, peptide composition, and secondary structural characteristics on the biological functions of PCOPs has not been comprehensively elucidated. Therefore, the development of collagen peptides from porcine skin represents a sustainable strategy to mitigate byproduct underutilization and environmental burden while providing a cost-effective source of bioactive ingredients for functional foods and skincare formulations.

To address these gaps, this study employed response surface methodology (RSM) to optimize the enzymatic hydrolysis of porcine skin collagen, with the primary objective of increasing the proportion of low-molecular-weight peptide fractions (<1000 Da), while using hydroxyproline content as a complementary quality indicator. The anti-aging-related and anti-melanogenic-related activities of the optimized PCOP were preliminarily evaluated using in vitro cell-based models. Furthermore, high-resolution peptidomics, in conjunction with circular dichroism spectroscopy, was applied to characterize peptide composition and secondary structural features, aiming to explore potential correlations within the peptide profile–conformation–bioactivity relationship. This work provides optimized process parameters and integrated structural insights, offering a focused scientific reference for the future investigation of PCOPs as potential functional peptide candidates.

## 2. Materials and Methods

### 2.1. Materials and Reagents

Porcine skin was obtained from Anhui Province, China. Fresh skin samples were cut into 1 cm × 1 cm pieces, pretreated with 2% (*w*/*v*) sodium bicarbonate at 65 °C for 30 min, thoroughly rinsed with deionized water, and stored at −20 °C until further use. Fish-derived collagen peptides were obtained from Rousselot (Bordeaux, France). The collagenase used in this study was developed in-house at the Biomass Engineering Research Institute, South China Agricultural University (Guangzhou, China). The enzyme preparation was a food-grade compound protease system mainly composed of neutral protease and alkaline protease, designed to provide broad-spectrum collagen-degrading activity. Enzymatic activity was assessed using type I collagen as the substrate, and one unit (U) of collagenase activity was defined as the amount of enzyme required to release 1 μg of free amino acids per minute under standard assay conditions (pH 7.5, 37 °C). Based on preliminary enzymatic characterization, the enzyme exhibited effective catalytic activity within a pH range of 5.5–8.5 and at temperatures of 45–65 °C, which was consistent with the hydrolysis conditions applied in this study. Therefore, enzymatic hydrolysis was optimized and controlled using enzyme dosage (U/g substrate) as the primary experimental variable.

1,1-Diphenyl-2-picrylhydrazyl (DPPH), tyrosinase (≥1000 U/mg, dry weight), kojic acid (≥99%), and Na_2_HPO_4_·12H_2_O (≥99%) were purchased from Macklin Biochemical Co., Ltd. (Shanghai, China). Fetal bovine serum (FBS) was obtained from Excell Biotechnology Co., Ltd. (Suzhou, China). Phosphate-buffered saline (PBS), Dulbecco’s Modified Eagle Medium (DMEM), and 0.25% trypsin and trypsin-EDTA solutions were purchased from Gibco (USA). α-Melanocyte-stimulating hormone (α-MSH) was purchased from MedChemExpress (Monmouth Junction, NJ, USA). L-3,4-dihydroxyphenylalanine (L-DOPA) and dimethyl sulfoxide (DMSO) were obtained from Sigma-Aldrich (St. Louis, MO, USA). Ferulic acid was sourced from Spec-Chem Industry Inc. (Nanjing, China). The β-galactosidase staining kit and MTT reagent were from Beyotime Biotechnology Co., Ltd. (Shanghai, China). Triton X-100 was sourced from Sangon Biotech Co., Ltd. (Shanghai, China). All other reagents were of analytical grade.

### 2.2. Preparation of Porcine Skin Collagen Oligopeptides

The substrate was sterilized and partially hydrolyzed by autoclaving at 121 °C for 30 min. The degree of hydrolysis (DH) was determined using the o-phthaldialdehyde (OPA) spectrophotometric method, with L-leucine as the standard. DH was calculated according to the ratio of free amino groups released to the total peptide bonds, as previously described. Autoclaving conditions were optimized until a DH of approximately 80%, which has been reported to be sufficient to disrupt the collagen triple-helix structure and enhance enzymatic accessibility. Distilled water was subsequently added according to the predetermined solid-to-liquid ratio, and the mixture was homogenized. Enzymatic hydrolysis was then performed under the optimized reaction conditions, after which the enzyme was inactivated by heating at 90 °C for 10 min.

To improve enzymatic hydrolysis efficiency and reduce potential off-flavors, sodium bicarbonate pretreatment was applied to adjust the substrate pH and partially unfold collagen structures, thereby enhancing enzyme accessibility. Distilled water was subsequently added according to the predetermined solid-to-liquid ratio, and the mixture was homogenized. Enzymatic hydrolysis was then performed under the optimized reaction conditions, after which the enzyme was inactivated by heating at 90 °C for 10 min.

Following cooling to room temperature, the hydrolysate was subjected to coarse and fine filtration using standard filter paper. To remove pigments and other chromophoric compounds that may affect product appearance and purity, the filtrate was decolorized by adding 3% (*w*/*w*) activated carbon and incubating at 65 °C for 30 min. The mixture was then centrifuged at 4000 rpm for 10 min, and the resulting supernatant was collected, freeze-dried, and stored at −20 °C until further use.

### 2.3. Response Surface Methodology Optimization

Based on the principles of the Box–Behnken design, the optimal combination of four independent variables—enzymatic hydrolysis temperature, pH, solid-to-liquid ratio, and enzyme dosage—was selected to optimize the preparation process of porcine skin collagen oligopeptides.

The design levels for the four factors in the RSM experiment were determined based on preliminary single-factor experiments, as shown in Table 1. Specifically, the temperature range (50 °C, 55 °C, 60 °C) and pH range (6.0, 6.5, 7.0) were selected to balance enzymatic activity and stability; enzyme dosage (0.5%, 0.7%, 0.9%, *w*/*w*) was chosen based on initial trials to ensure sufficient cleavage without excessive enzyme use; and solid-to-liquid ratio (1:2, 1:3, 1:4) was set to maintain adequate substrate dispersion and enzyme-substrate interaction. These ranges ensured that the response surface captured the key factors affecting the proportion of collagen oligopeptides with molecular weight < 1000 Da while remaining industrially feasible.

A four-factor Box–Behnken experimental design was employed, with experiments conducted at 30 experimental points. The data were analyzed using a response surface regression program. The software Design Expert (Version 8.05b, Stat-Ease, Minneapolis, MN, USA) was used to derive a second-order polynomial regression equation for the content of collagen oligopeptides with a molecular weight < 1000 Da. Freeze-dried PCOP powder was prepared under the determined optimal experimental conditions and stored at −20 °C for further analysis.

**Table 1:** Response surface design test results.

Run	A: Substrate Concentration (%)	B: Temperature (°C)	C: pH	D: Solid-to-Liquid Ratio	Molecular Weights Below 1000 Da (%)
1	0.7	55	7	4	84.19
2	0.5	50	6.5	3	82.26
3	0.7	55	6.5	3	84.24
4	0.9	50	6.5	3	84.63
5	0.7	55	6	4	83.05
6	0.7	55	6.5	3	84.87
7	0.7	55	7	2	86.62
8	0.9	60	6.5	3	84.31
9	0.5	60	6.5	3	81.10
10	0.7	55	6	2	82.40
11	0.5	55	6.5	2	83.15
12	0.7	55	6.5	3	84.91
13	0.7	55	6.5	3	85.47
14	0.7	50	6	3	83.92
15	0.7	60	6	3	81.42
16	0.9	55	6.5	4	85.52
17	0.7	60	7	3	85.20
18	0.9	55	6.5	2	86.27
19	0.7	50	7	3	85.22
20	0.5	55	6.5	4	83.79
21	0.7	55	6.5	3	85.00
22	0.7	60	6.5	2	83.02
23	0.5	55	7	3	83.52
24	0.7	50	6.5	4	83.73
25	0.9	55	6	3	83.32
26	0.7	60	6.5	4	82.86
27	0.7	50	6.5	2	84.47
28	0.9	55	7	3	85.67
29	0.7	55	6.5	3	83.89
30	0.5	55	6	3	80.21

### 2.4. Determination of Molecular Weight Distribution

The molecular weight distribution of porcine collagen oligopeptides was determined according to the method reported by Lin et al. [26] High-performance gel permeation chromatography (GPC) was employed to analyze the molecular weight distribution of porcine collagen oligopeptides. Separation was performed using a TSKgel G2000 SWXL column (300 × 7.8 mm) with a mobile phase of acetonitrile-water-trifluoroacetic acid (45:55:0.1, *v*/*v*/*v*), flow rate of 0.5 mL/min, column temperature of 30 °C, and detection wavelength of 220 nm. Molecular weight standards were cytochrome C (12,500 Da), bacitracin (1422.69 Da), Gly-Gly-Tyr-Arg (451.49 Da), and Gly-Gly-Gly (189.17 Da). Samples and standards were filtered through a 0.22 μm membrane before injection. A standard curve was plotted with retention time on the *x*-axis and logarithm of molecular weight on the *y*-axis (y = −0.2232x + 6.798, R^2^ = 0.9980). Sample molecular weights and their distributions were calculated using LabSolutions GPC software, Version 5.121 (Shimadzu Corporation, Kyoto, Japan). 

### 2.5. Determination of Hydroxyproline Content

The hydroxyproline content in PCOP was determined using a modified version of the method described by Yoni [27]. The sample was hydrolyzed with sulfuric acid at 105 °C for 16 h. The hydrolysate was then filtered and diluted. The product was oxidized with chloramine T and reacted with 4-Dimethylaminobenzaldehyde to form a red compound, the absorbance of which was measured at 558 nm. A standard curve was generated by plotting the absorbance of the standard working solutions (with the blank subtracted) on the *y*-axis against the corresponding concentrations on the *x*-axis (c = 0.2032x + 0.0054, x = absorbance, R^2^ = 0.9992).

### 2.6. In Vitro Assay of H_2_O_2_-Induced Cellular Senescence

The method was adapted and modified from Qin et al. [28]. Human embryonic skin fibroblasts (ESF-M) in good growth condition and at the mid-to-late logarithmic growth phase were collected, counted, and seeded at an adjusted density into 24-well plates, followed by incubation for 18–24 h, after which the medium was replaced with the corresponding treatment medium for a further 24 h. After PBS washing, all groups except the normal control were treated with maintenance medium containing 300 μM H_2_O_2_ for 24 h to induce oxidative stress-mediated cellular senescence. The medium was then replaced with complete medium for continued culture for 72 h, followed by cell fixation, overnight incubation with β-galactosidase staining working solution, and nuclear counterstaining. Bright-field images and corresponding nuclear fluorescence images were captured under a microscope. For each group, senescent cells were quantified from at least three randomly selected fields of view, and the positive rate was calculated.

Positive Rate (%) = (Number of Positive Cells/Total Number of Cells) × 100

### 2.7. Cytotoxicity Assay on B16 Cells

The method was modified from Chen et al. [29]. B16 cells at 80–90% confluence were collected and seeded at a density of 5 × 10^4^ cells/well (96-well plate) or 2 × 10^5^ cells/well (6-well plate). All treatments were conducted in triplicate, and experiments were independently repeated at least three times. Kojic acid (50 μM) was used as a positive control for tyrosinase inhibition, followed by incubation for 24 h. After 24 h of culture, the medium was replaced with complete medium containing different concentrations of the sample and continue culturing for another 24 h. Add MTT solution and incubate in the dark for 2 h. Discard the supernatant, add DMSO, and shake to dissolve for 10 min. Measure the absorbance of each well at a wavelength of 570 nm.

Viability (%) = (OD_570e_/OD_570b_) × 100

OD_570e_ is the absorbance of the experimental sample group or positive control group, OD_570b_ is the absorbance of the blank control group.

### 2.8. Tyrosinase Activity Assay in B16 Cells

The method was modified from Hu et al. [21]. B16 cells were seeded into 6-well plates and incubated for 24 h. The culture medium was then replaced with fresh medium containing different concentrations of the sample and α-MSH, followed by incubation for an additional 72 h. The medium was discarded, cells were washed with PBS, and Triton X-100 was added to lyse the cells. Following freeze–thaw cycles at −80 °C and centrifugation at 4 °C, the supernatant was collected. The cell supernatant was reacted with L-DOPA solution at 37 °C in the dark for 2 h, and absorbance was measured at 490 nm.

Relative Tyrosinase Activity (%) = (OD_490e_/OD_490b_) × 100

OD_490e_ is the absorbance of the experimental sample group or positive control group, OD_490b_ is the absorbance of the model control group.

### 2.9. Melanin Synthesis Assay in B16 Cells

The method was modified from Hu et al. [21]. After incubating B16 cells for 24 h, replace the medium with fresh medium containing different concentrations of the sample and α-MSH, and continue culturing for 72 h. Discard the medium, wash with PBS, collect the cells, and centrifuge. Add NaOH solution, heat in an 80 °C water bath for 1.5 h, and measure the absorbance at 405 nm.

Relative Melanin Content (%) = (OD_405e_/OD_405b_) × 100

OD_405e_ is the absorbance of the experimental sample group or positive control group, and OD_405b_ is the absorbance of the model control group.

### 2.10. Peptidomic Identification and Differential Analysis

Peptide separation was performed using a nano-scale liquid chromatography system (Thermo Scientific, San Jose, CA, USA). The column used was a Thermo Scientific EASY column (ES906, 15 cm × 150 μm, 2 μm; Thermo Scientific, San Jose, CA, USA). Mobile phase A consisted of 0.1% formic acid aqueous solution, while phase B comprised 0.1% formic acid-80% acetonitrile solution. The flow rate was 800 nL/min. The separated fractions were subjected to data-dependent acquisition (DDA) acquisition on an Astral high-resolution mass spectrometer in positive ion mode, with a scan range of 380–980 *m*/*z*, resolution of 240,000 (at 200 *m*/*z*), and HCD fragmentation energy of 25 eV. Mass spectrometry data were processed using PEAKS software (Version 13.1). Database searching was performed against the UniProt Sus scrofa protein database. Peptide-spectrum matches (PSMs) were filtered using a false discovery rate (FDR) of 1% at both peptide and protein levels. Only peptides with −10lgP ≥ 20 and proteins identified with at least one unique peptide were retained. Label-free quantification was performed using PEAKS software (Version 13.1) with a mass tolerance of 20 ppm and retention time tolerance of 1.0 min.

### 2.11. Circular Dichroism Analysis

Following the method of Huina et al. [30], the sample was dissolved to prepare a test solution at a concentration of 0.2 mg/mL. Using a circular dichroism spectrometer (JASCO J-1500; JASCO, Tokyo, Japan), the sample was scanned at 24.7 °C over a wavelength range of 190–260 nm (with a 1 mm path length in the cuvette) to obtain the circular dichroism spectrum. After smoothing the raw data using Spectra Analysis software (Version not specified, provided by Shiyanjia/Scientific Compass platform, Hangzhou, China), analyze and calculate the relative content of the protein’s secondary structure via the DichroWeb platform.

### 2.12. Statistical Analysis

All data were expressed as mean ± standard deviation (SD). Statistical analysis was performed using GraphPad Prism 9.0. Normality was assessed using the Shapiro–Wilk test, and homogeneity of variances was evaluated using Levene’s test.

Comparisons among multiple groups were conducted using one-way analysis of variance (ANOVA) followed by Tukey’s post hoc test. A value of *p* < 0.05 was considered statistically significant.

## 3. Results and Discussion

### 3.1. Model Regression and Analysis of Variance

The data analysis (Table 2) revealed significant variations in the content of PCOP < 1000 Da under different experimental conditions. Based on the experimental data and parameter estimation, the second-order polynomial regression equation describing the effects of four factors on the proportion of PCOP with molecular weight < 1000 Da was established as follows:

Y = 84.73 + 1.31A − 0.5274B + 1.34C − 0.2318D + 0.2096AB − 0.2416AC − 0.3483AD + 0.6218BC + 0.1451BD − 0.7699CD − 0.6402A^2^ − 0.8388B^2^ − 0.5156C^2^ + 0.0254D^2^

The model exhibited good goodness-of-fit, with a high coefficient of determination (R^2^ = 0.9735) and adjusted R^2^ of 0.9450, while the lack-of-fit test was not statistically significant (*p* = 0.9774). These results suggest that the model has reliable predictive capability within the experimental domain. The magnitude of the regression coefficients indicated the following order of effect on the proportion of PCOP < 1000 Da: pH > enzyme dosage > hydrolysis temperature > solid-to-liquid ratio. Molecular weight distribution was prioritized as the main optimization criterion due to its relevance to bioavailability and functional activity. However, factors such as yield, cost, and scalability were not included in this optimization and should be considered in future developments.

### 3.2. Analysis of Response Surface Interactions and Process Validation

**Figure 1:**
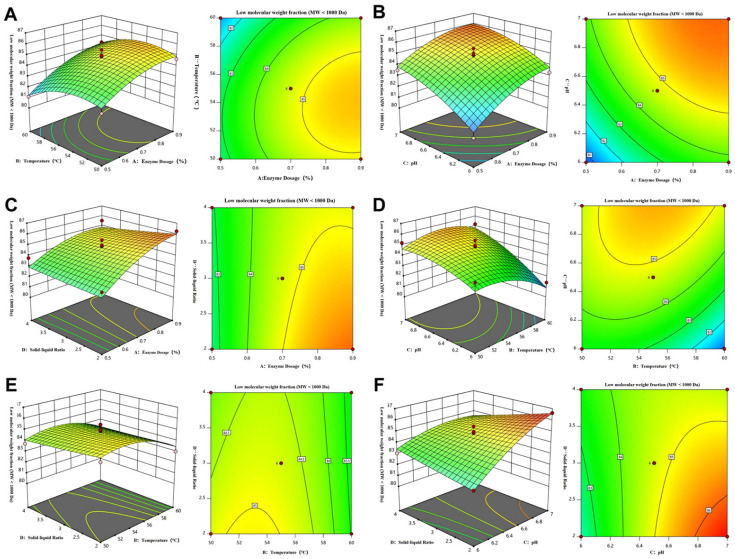
Contour plot response surfaces and corresponding contour plots of collagen oligopeptide < 1000 Da content of porcine skin as affected by each factor under different factor interactions. (**A**). Interaction between enzyme addition and temperature. (**B**). Interaction between enzyme addition and pH. (**C**). Interaction between enzyme addition and solid–liquid ratio. (**D**). Interaction between temperature and pH. (**E**). Interaction between temperature and solid–liquid ratio. (**F**). Interaction between pH and solid–liquid ratio.

Increasing enzyme dosage and pH significantly increased the proportion of PCOP < 1000 Da (Figure 1A–C or Figure 1F), consistent with the model’s prediction. As enzyme concentration increased, collagen macromolecules were cleaved more efficiently into shorter peptides, increasing the proportion of low-molecular-weight peptides.

This trend is likely due to the increased availability of catalytic sites at higher enzyme concentrations, enhancing peptide bond cleavage and promoting the formation of smaller peptides [31]. Under constant enzyme concentration and temperature, the fraction of collagen oligopeptides < 1000 Da increased with rising pH (Figure 1B or Figure 1D or Figure 1F). This observation may be related to the catalytic characteristics of collagenases, some of which have been reported to belong to the metalloproteinase family and may rely on metal ion coordination (e.g., Zn^2+^) at their active sites. A mildly alkaline environment may enhance the coordination between the metal ion cofactors and substrate, while stabilizing the enzyme’s tertiary structure, thereby improving catalytic efficiency [32].

Temperature had a bidirectional effect on product distribution. Within the optimal range of 50–55 °C, increasing temperature enhanced the collision frequency between enzyme and substrate molecules, accelerating hydrolysis. However, when the temperature exceeded this range, partial destabilization of the enzyme’s structures likely occurred, reducing catalytic efficiency and decreasing the proportion of collagen oligopeptides < 1000 Da (Figure 1D,E).

In contrast, the solid-to-liquid ratio had a minor influence on the yield of collagen oligopeptides < 1000 Da (Figure 1C or Figure 1E,F). Within the tested range, variations in substrate concentration had little effect on the collision probability between enzyme and substrate molecules, contributing marginally to the generation of low-molecular-weight peptides [33].

Under optimized conditions (solid-to-liquid ratio 1:2, 52.3 °C, enzyme loading 0.9%, and pH 7.0), the proportion of collagen oligopeptides < 1000 Da reached 85.77 ± 0.63%, closely matching the model prediction (87.03%, *p* > 0.05). This agreement validates the reliability and predictive capability of the response surface model. These results demonstrate the optimization of molecular weight distribution under the selected conditions; however, further studies are warranted to integrate additional industrially relevant parameters to facilitate practical application at larger scales.

### 3.3. Molecular Weight Distribution and Hydroxyproline Content of Porcine Skin Collagen Oligopeptides

The molecular weight distribution and hydroxyproline content of PCOP, with clarified molecular weight subranges and their summation, are presented in Table 3. The molecular weight profile reflects the degree of collagen degradation during enzymatic hydrolysis and the physicochemical characteristics of the resulting peptides. Previous studies have shown that low-molecular-weight collagen peptides (<1000 Da) are more readily absorbed by the intestinal epithelium and are often associated with improved bioavailability and certain biological activities [34]. Therefore, analysis of the molecular weight distribution provides a basis for optimizing hydrolysis conditions and serves as an indicator for assessing the functional potential of the product. In addition, hydroxyproline, a collagen-specific amino acid, is commonly used as a parameter for evaluating the collagen content of hydrolysates [35]. Previous studies have also suggested that hydroxyproline may serve as an auxiliary biochemical indicator when evaluating collagen-derived preparations [36].

Collagen peptides with relatively higher hydroxyproline content have been reported to be associated with fibroblast activation and collagen-related cellular responses [21]. The PCOP obtained in this study contained 9.68 ± 0.16% hydroxyproline, indicating that the enzymatic hydrolysis process preserved a substantial proportion of collagen-derived peptide components. This compositional feature provides structural context for subsequent evaluation of the biological activities of PCOP.

The digestion and absorption of collagen peptides in humans constitute a complex and finely regulated process. Orally ingested macromolecular collagen generally requires degradation into amino acids or small peptides within the gastrointestinal tract prior to absorption [22]. In contrast, low-molecular-weight collagen oligopeptides, particularly those below 1000 Da, can be absorbed by intestinal epithelial cells, in part as intact small peptides, which may contribute to improved bioavailability [37]. Consequently, a lower molecular weight is generally associated with higher intestinal absorption efficiency and bioavailability [38]. GPC analysis revealed that the molecular weight of PCOP was predominantly distributed below 1000 Da (85.77%), with the major fraction (48.56%) ranging between 180 and 500 Da. Peptides within 500–1000 Da constituted the second largest fraction (29.31%). Notably, peptide segments within the 180–500 Da range have been reported to be capable of intestinal absorption and may participate in biological processes as signaling or structural components. This molecular weight distribution shows a higher proportion of low-molecular-weight fractions than that reported for many previously studied collagen hydrolysates derived from other sources (e.g., porcine skin 28.9%, bovine bone 55.1%, yellowfin tuna skin 60%) [39,40], indicating that the composite collagenase system applied in this study favored the generation of a higher proportion of low-molecular-weight peptides under the tested conditions.

Unlike conventional single-enzyme systems, the composite collagenase developed here was generated through multi-strain co-fermentation and multi-factor synergistic enhancement, resulting in oligopeptides with smaller average molecular weights and a more concentrated distribution. These findings suggest that enzyme synergy contributed to efficient molecular-level cleavage and provide a basis for subsequent investigation of the functional properties and biological activities of the resulting collagen oligopeptides. Furthermore, they substantiate the significant process advantages of the enzymatic hydrolysis strategy employed in this study, enabling the efficient preparation of collagen oligopeptides with enhanced bioactivity.

### 3.4. Inhibitory Effect of PCOP on H_2_O_2_-Induced Senescence in Fibroblasts

**Figure 2:**
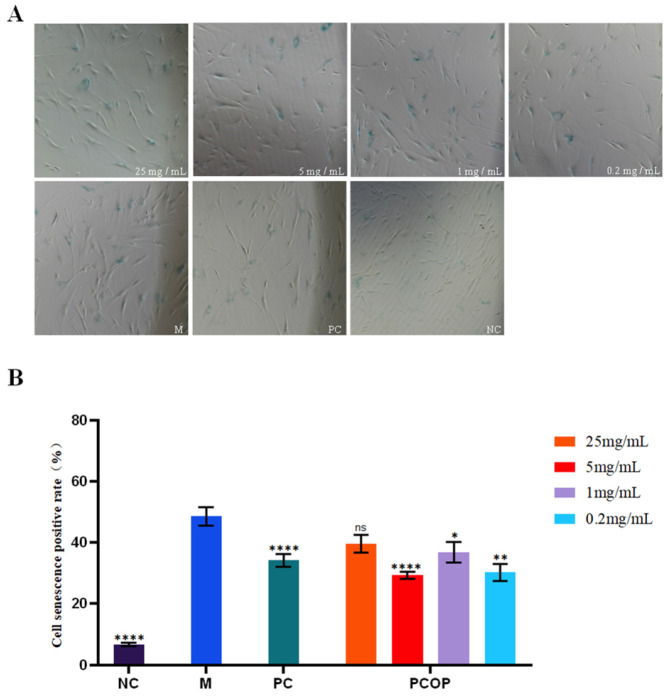
Inhibitory effects of different concentrations of PCOP on H_2_O_2_-induced senescence in ESF-M. (**A**). Cell senescence staining map. (**B**). Positive rate of cellular senescence. NC: blank control group; M: model group; PC: positive control group. There were significant differences among different concentrations (*p* < 0.05). All data are presented as the mean ± SD. ns = no significant (*p* > 0.05), * *p* < 0.05, ** *p* < 0.01, **** *p* < 0.0001.

In the presence of intracellular Fe^2+^, hydrogen peroxide (H_2_O_2_) is converted into highly reactive hydroxyl radicals via the Fenton reaction. This process induces oxidative damage to intracellular biomolecules—including DNA, proteins, and lipids—thereby accelerating cellular oxidative stress and ultimately leading to cell death [41]. Accordingly, H_2_O_2_ is widely employed as an inducer of oxidative stress in in vitro senescence models. The effects of PCOP at different concentrations on H_2_O_2_-induced senescence in ESF-M cells are presented in Figure 2. Compared with the model group, PCOP treatment reduced the proportion of senescence-positive cells in a concentration-dependent manner under H_2_O_2_-induced oxidative stress. Among the tested concentrations, 5 mg/mL PCOP resulted in the greatest reduction in the proportion of senescence-positive cells, decreasing the senescence-positive rate from 48.96% to 29.30% (a 39.66% decrease, *p* < 0.05). The observed reduction in cellular senescence may be associated with the Pro/Hyp-rich peptide composition of PCOP. The potential involvement of signaling pathways such as MAPK or TGF-β is hypothesized based on previous reports, although these pathways were not directly investigated in the present study [42]. Furthermore, the observed nonlinear dose–response relationship (e.g., 1 mg/mL elicited a weaker effect than 0.2 mg/mL, *p* > 0.05) suggests that the cellular response to PCOP may be influenced by concentration-dependent effects, potentially involving signaling saturation, peptide–peptide interactions within complex mixtures, or limitations in cellular uptake. It should be noted that all biological evaluations in this study were conducted using in vitro cell models, and the concentrations applied may not directly reflect physiological exposure levels in vivo.

### 3.5. Cytotoxic Effects of the Samples on B16 Cells

**Figure 3:**
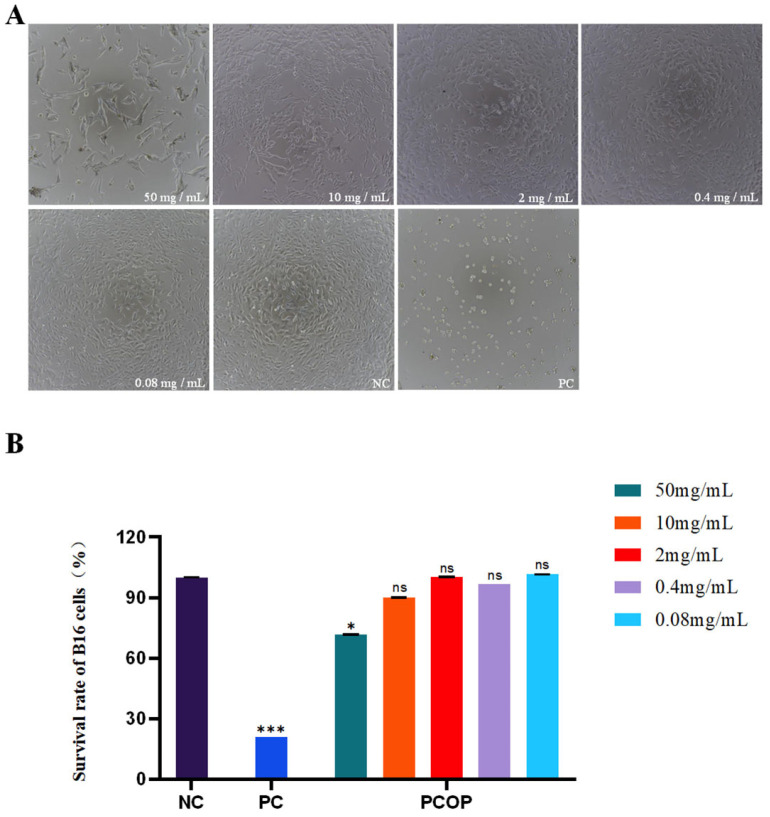
Effects of different concentrations of collagen peptides on the viability of B16 cells. (**A**). Representative microscopic images of B16 cells. (**B**). Cell viability determined by the MTT reagent assay. NC: blank control group; PC: positive control group. There were significant differences among different concentrations (*p* < 0.05). All data are presented as the mean ± SD. ns = no significant (*p* > 0.05), * *p* < 0.05, *** *p* < 0.001.

Cytotoxicity assays are commonly used to evaluate the effects of test samples on cell viability and to define appropriate concentration ranges for subsequent cell-based functional studies. Cell viability was used as an indicator to assess the effects of PCOP on B16 cells [29]. The experimental results are presented in Figure 3. As shown in Figure 3A, microscopic examination revealed that cells in the negative control group exhibited vigorous growth with characteristic spindle-shaped or polygonal morphology, whereas cells in the positive control group displayed markedly reduced density and evident morphological deterioration.

Cells treated with different concentrations of PCOP retained an overall intact morphology without obvious morphological features indicative of cell death. Quantitative analysis (Figure 3B) showed that PCOP exerted no marked cytotoxic effects on B16 cells, with a CV_90_ (concentration maintaining 90% cell viability) of 14.32 mg/mL. At concentrations below the CV_90_, cell viability consistently remained above 90%, and even at the highest tested concentration (50 mg/mL), viability was approximately 70%. These results indicate that PCOP showed limited cytotoxic effects toward B16 cells within the tested concentration range, supporting the selection of appropriate concentrations for subsequent anti-melanogenic assays.

### 3.6. Effect of the Samples on Tyrosinase Activity in B16 Cells

Tyrosinase is a multifunctional, copper-dependent oxidoreductase predominantly expressed in melanocytes. Together with its related isoenzymes, tyrosinase-related protein 1 (TYRP1) and tyrosinase-related protein 2 (TYRP2), tyrosinase catalyzes the sequential oxidation of tyrosine during melanin biosynthesis and plays a central role in melanogenesis [43,44]. PCOP exhibited a concentration-dependent inhibitory effect on intracellular tyrosinase activity in α-MSH-stimulated B16 cells within the tested range (0.2–10 mg/mL). Significant inhibition was observed at 1 and 5 mg/mL (*p* < 0.01), and the inhibitory effect at 10 mg/mL was comparable to that observed for the positive control, kojic acid, under the same experimental conditions., which was used as a reference tyrosinase inhibitor. The observed inhibitory effect may be associated with enzyme-peptide interactions, such as potential copper ion chelation or interference with enzyme conformation, as suggested by previous studies on collagen-derived peptides [45]. In addition, because tyrosinase activity is sensitive to the cellular redox state and collagen-derived peptides have been reported to exhibit antioxidant properties, PCOP may indirectly modulate tyrosinase activity through redox-related mechanisms. Collectively, these findings indicate that PCOP suppresses tyrosinase activity at the cellular level, although the underlying molecular mechanisms remain to be elucidated. It should be noted that all biological evaluations in this study were conducted using in vitro cell models, and the concentrations applied may not directly correspond to physiological exposure levels in vivo.

### 3.7. Effect of the Samples on Melanin Synthesis in B16 Cells

**Figure 4:**
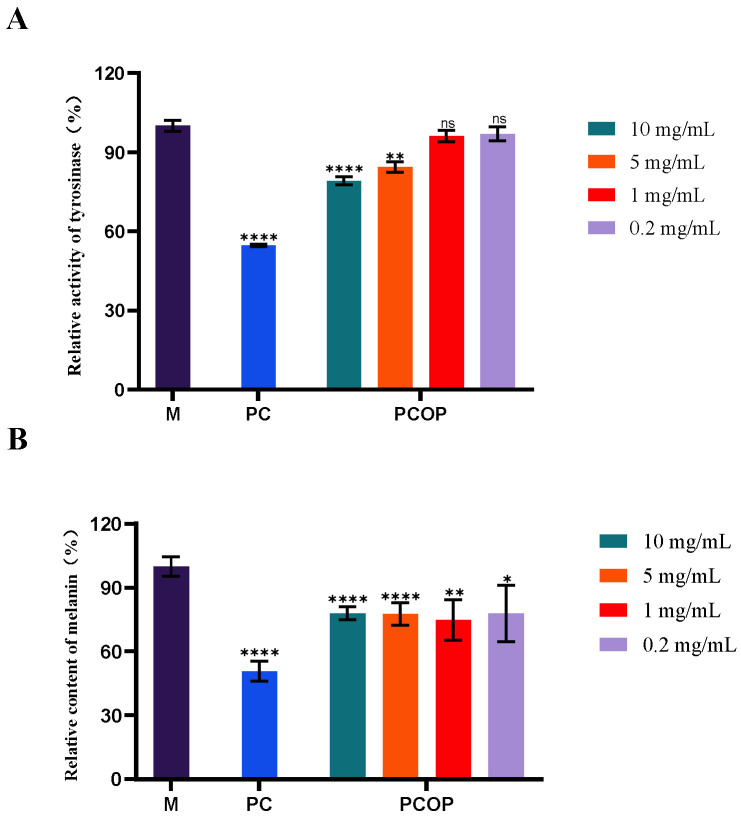
Effects of PCOP at different concentrations on tyrosinase activity and melanin production in B16 cells. (**A**). Effect of PCOP on intracellular tyrosinase activity in B16 cells. (**B**). Effect of PCOP on melanin content in α-MSH-stimulated B16 cells. M: α-MSH-stimulated model group; PC: positive control group. Statistical differences were observed among different treatment concentrations (*p* < 0.05). All data are presented as the mean ± SD. ns = no significant (*p* > 0.05), * *p* < 0.05, ** *p* < 0.01, **** *p* < 0.0001.

Melanin is the primary pigment responsible for the coloration of human skin, eyes, and hair. Melanin synthesis is a stress-responsive process in melanocytes, and its dysregulation is associated with various melanogenic disorders, including freckles, lentigines, age spots, and malignant melanoma [33]. As shown in Figure 4B, PCOP significantly reduced α-MSH-induced melanin production in B16 cells within the tested concentration range (0.2–10 mg/mL, *p* < 0.05). At 1.0 mg/mL, melanin content decreased to 74.80 ± 9.60% relative to the model group, which is comparable to the inhibition reported for grass carp scale collagen peptides at similar concentration ranges [20]. In addition to direct tyrosinase inhibition, PCOP may influence melanogenesis through modulation of the cAMP/PKA/CREB/MITF signaling pathway, as suggested by previous studies, although this mechanism was not directly examined in the present work [46,47].

### 3.8. Peptidomic and Structural Analysis

To further investigate the structure–activity relationship of PCOP, peptidomic profiling was combined with circular dichroism (CD) spectroscopy. Comparative analyses were conducted using a commercial fish collagen peptide (Rousselot) to systematically characterize amino acid composition, peptide molecular weight distribution, and secondary structural features, providing descriptive insights into potential associations between peptide properties, secondary structures, and observed biological activities.

#### 3.8.1. Peptidomic Feature Analysis

**Figure 5:**
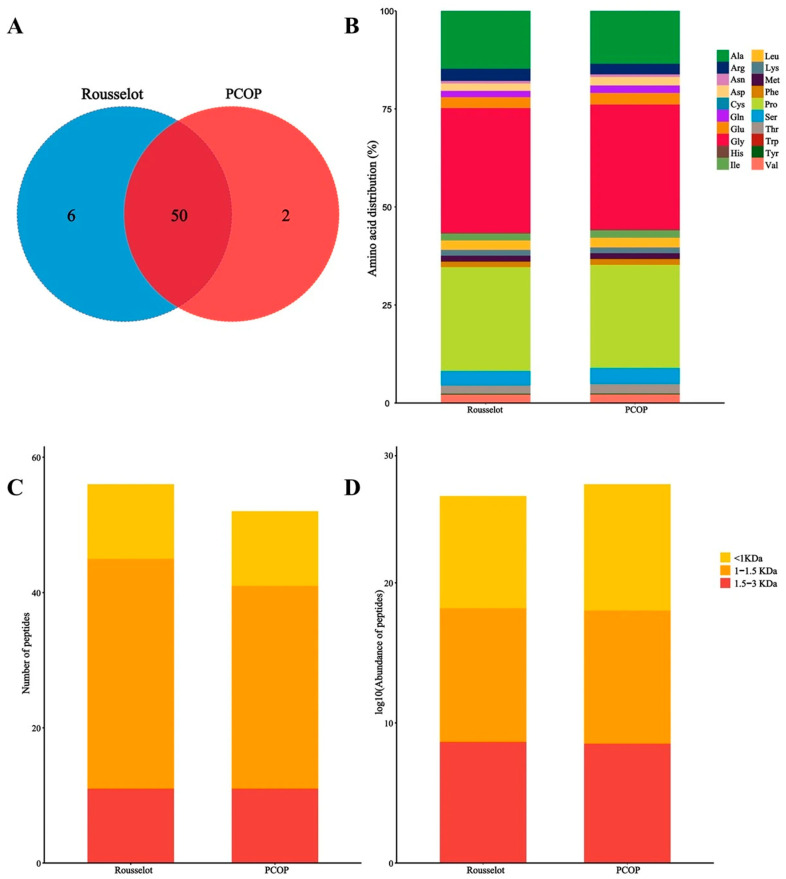
Comparative analysis of PCOP and Rousselot peptidomics. (**A**). Venn diagram of peptide segment. (**B**). Amino acid composition ratio chart. (**C**). The number of peptides in different molecular weight intervals. (**D**). Polypeptides in different molecular weight intervals.

Using data-dependent acquisition (DDA) mass spectrometry, a total of 58 distinct peptide sequences corresponding to 107 proteins were identified in PCOP. As shown in Figure 5A, two peptide fragments—GLTGPIGPPGPA and GDSGAPGPAGPTGAPGPQ—were uniquely detected in PCOP but absent in the Rousselot sample, which may contribute to its distinct biological profile. Although classical collagen-derived bioactive motifs are difficult to quantify within complex peptide mixtures, their presence is generally associated with collagen peptide-mediated anti-aging effects, such as stimulation of fibroblast proliferation and extracellular matrix synthesis [48]. Amino acid composition analysis (Figure 5B) indicated that both PCOP and Rousselot peptides were enriched in glycine (Gly), proline (Pro), and alanine (Ala), consistent with the characteristic (Gly-X-Y)_n_ repeating motif of collagen. Both preparations were predominantly composed of low-molecular-weight fractions (<3000 Da), with the highest abundance in the <1500 Da range (Figure 5C,D). Specifically, 47.64% of peptides were distributed within 180–500 Da, and 28.34% within 500–1000 Da. These distributions were consistent with gel permeation chromatography (GPC) results, confirming the reproducibility and accuracy of molecular weight characterization.

The agreement between GPC and peptidomic data further supports that PCOP peptides possess physicochemical properties favorable for gastrointestinal absorption and, potentially, transdermal delivery, which may contribute to systemic bioavailability and biological efficacy. The peptidomic analysis revealed that PCOP is enriched in Pro-Gly motifs and predominantly composed of low-molecular-weight peptides (<1000 Da). Previous studies have suggested that Pro-Gly-rich peptides may stimulate fibroblast proliferation and support extracellular matrix synthesis, while small peptide size can facilitate intestinal absorption and potentially transdermal delivery [20,21]. Together, these features may plausibly contribute to the observed cellular effects in vitro, although direct causality was not experimentally validated.

#### 3.8.2. Peptide Source and Functional Inference

**Figure 6:**
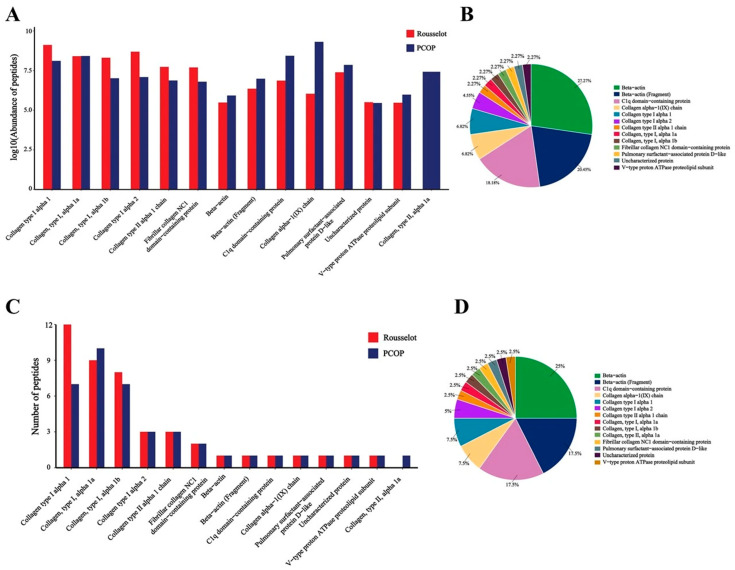
Statistics of the main source proteins of PCOP and Rousselot polypeptides. (**A**). Peptide abundance corresponding to protein. (**B**). The proportion of polypeptides contributed by each protein in Rousselot. (**C**). The number of polypeptides corresponding to proteins. (**D**). The proportion of polypeptides contributed by each protein in PCOP. Values are rounded to the nearest percent; totals may not sum to exactly 100%.

Figure 6 shows the precursor proteins corresponding to the 15 most abundant peptides identified in PCOP and the Rousselot reference. In PCOP, the majority of peptides were derived mainly from collagen type I alpha-1 chain (COL1A1), collagen type I alpha-2 chain (COL1A2), and β-actin. This distribution suggests that the enzymatic hydrolysis process preserves fragments from key parent proteins. Collagen plays a central role in maintaining dermal structural integrity, while actin is involved in cytoskeletal organization and cellular dynamics related to extracellular matrix (ECM) remodeling. Consequently, these derived peptides may retain functional properties relevant to fibroblast proliferation and collagen network maintenance, indicating their potential utility in dermal anti-aging and whitening applications [49].

#### 3.8.3. Secondary Structure Analysis

**Figure 7:**
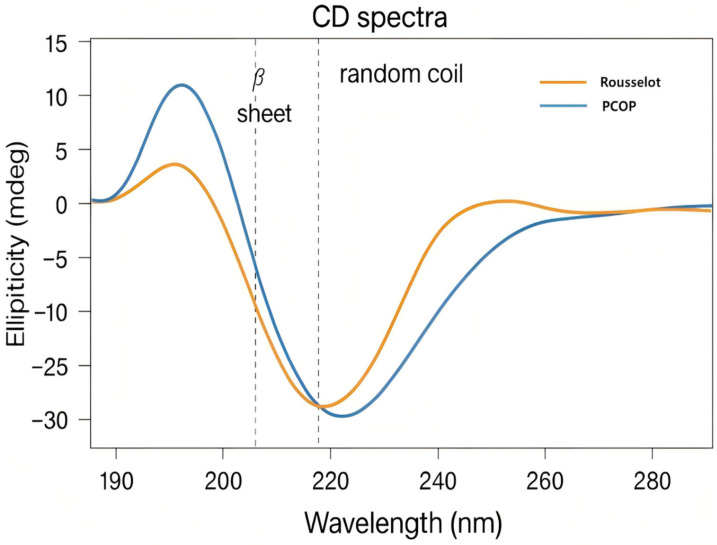
Circular dichroism spectral analysis of the purified PCOP and Rousselot.

CD spectroscopy was used to assess the secondary structural characteristics of PCOP, based on the characteristic absorption signals in the far-ultraviolet (far-UV) region. As shown in Figure 7 and Table 4, the secondary structure of PCOP comprised primarily β-sheets (35.3%) and random coils (38.9%), with a minor α-helix content (5.5%). These results indicate that the native triple-helix structure of collagen was largely disrupted during enzymatic hydrolysis, producing peptides with a more flexible and disordered conformation.

The combination of flexible random coils and relatively stable β-sheets may enable the peptides to adopt conformations that facilitate interactions with enzyme active sites or cell surface receptors, potentially influencing biological outcomes such as tyrosinase inhibition. While these structural properties offer a plausible mechanistic explanation, they remain predictive and do not establish direct cause-and-effect relationships in the cellular assays. The random coil-rich conformation could contribute to functional activity, although the CD spectra reflect the ensemble-averaged properties of a heterogeneous peptide mixture. This structural flexibility may allow peptide chains to overcome steric hindrance, facilitating interactions with three-dimensional enzyme active sites or cell surface receptors [45]. In the context of melanogenesis inhibition, for example, tyrosinase is a copper-containing metalloenzyme with a relatively narrow active site. The flexible peptides in PCOP may bind to copper ions or key residues (e.g., histidine) within the active site, potentially reducing catalytic activity. This provides a plausible structural explanation for the tyrosinase inhibition observed in B16 cells, without asserting direct causality [50]. Additionally, β-sheet content is associated with peptide stability and participation in self-assembly or biomolecular interactions [32]. The relatively high proportion of β-sheets in PCOP may enhance local structural stability, possibly affecting peptide behavior in specific microenvironments, although resistance to enzymatic degradation was not directly tested. This property may potentially contribute to improved structural stability and functional persistence in biological environments, although in vivo half-life was not evaluated in this study.

## 4. Conclusions

This study applied response surface methodology to optimize the enzymatic preparation of PCOP, resulting in a preparation with a predominance of low-molecular-weight (<1000 Da) components (85.77% by weight) and an elevated hydroxyproline content (9.68%). A systematic association was observed between the biological effects of PCOP at the cellular level (anti-aging and anti-melanogenic activity) and its molecular characteristics, including peptide composition and secondary structure. Peptidomic analysis revealed a characteristic peptide fingerprint containing segments enriched in the Gly-Pro-X motif. Structurally, PCOP exhibited a flexible and dynamic conformation in solution, primarily composed of β-sheets (35.3%) and random coils (38.9%). These molecular features may contribute to the observed biological effects, offering a plausible mechanistic context without asserting direct causality.

Overall, this work provides a practical enzymatic strategy and a molecular characterization framework for the utilization of porcine skin as a collagen source. Further research should focus on: (1) isolating and identifying specific peptide segments responsible for bioactivity and validating their individual effects; (2) assessing in vivo efficacy, absorption, and metabolism of PCOP in animal models; and (3) applying transcriptomic or proteomic approaches to clarify molecular signaling pathways involved in cellular aging and melanogenesis. Such investigations will support the rational development of PCOP and further understanding of its potential contributions to skin health.

## Figures and Tables

**Table 2:** Analysis of variance of regression model.

Source	Sum of Squares	DOF	Mean Square	F Value	*p* Value
Model	58.94	14	4.21	34.14	<0.0001 **
A	20.51	1	20.51	166.33	<0.0001 **
B	3.34	1	3.34	27.07	0.0002 **
C	21.61	1	21.61	175.28	<0.0001 **
D	0.6449	1	0.6449	5.23	0.0396 *
A^2^	2.81	1	2.81	22.80	0.0004 **
B^2^	4.82	1	4.82	39.13	<0.0001 **
C^2^	1.82	1	1.82	14.78	0.0020 **
D^2^	0.0044	1	0.0044	0.0359	0.8527
AB	0.1756	1	0.1756	1.42	0.2540
AC	0.2335	1	0.2335	1.89	0.1920
AD	0.4853	1	0.4853	3.94	0.0688
BC	1.55	1	1.55	12.54	0.0036 **
BD	0.0842	1	0.0842	0.6830	0.4235
CD	2.37	1	2.37	19.23	0.0007 **
Residual	1.60	13	0.1233		
Lack of Fit	0.6413	10	0.0641	0.2000	0.9774
Pure Error	0.9617	3	0.3206		
Cor Total	65.19	29			

* Significant difference, *p* < 0.05; ** Very significant, *p* < 0.01. Coefficient of determination, R^2^ = 0.9735, Adjustment coefficient, R^2^Adj = 0.9450.

**Table 3:** Molecular weight distribution and hydroxyproline content of porcine skin collagen oligopeptides.

Ingredient	Content (%)
Hydroxyproline Content	9.68 ± 0.16
Molecular weight ranges < 180 Da	7.9
Molecular weight ranges between 180~500 Da	48.56
Molecular weight ranges between 500~1000 Da	29.31
Subtotal (<1000 Da)	85.77
Molecular weight > 1000 Da	14.23

**Table 4:** Secondary Structure Content of PCOP and Rousselot Peptides Based on CD Spectroscopy.

Sample	α-Helix (%)	β-Sheet (%)	β-Turn (%)	Random Coil (%)
PCOP	5.5	20.3	35.3	38.9
Rousselot	6.8	33.8	22.1	37.3

## Data Availability

The original contributions presented in this study are included in the article. Further inquiries can be directed to the corresponding authors.

## References

[1] Varani J., Dame M.K., Rittie L., Fligiel S.E.G., Kang S., Fisher G.J., Voorhees J.J. (2006). Decreased collagen production in chronologically aged skin—Roles of age-dependent alteration in fibroblast function and defective mechanical stimulation. Am. J. Pathol..

[2] Zhang S.B., Duan E.K. (2018). Fighting against Skin Aging: The Way from Bench to Bedside. Cell Transplant..

[3] Zhou Y.J., Jiang Y.F., Zhang Y., Yokoyama W., Wu J.P., Chang S.K.C., Hong H., Luo Y.K., Li B., Tan Y.Q. (2025). Unveiling a new chapter for collagen peptides: Comprehensive insights into oral bioavailability and the enhancement via encapsulation systems. Trends Food Sci. Technol..

[4] Hong H., Fan H.B., Chalamaiah M., Wu J.P. (2019). Preparation of low-molecular-weight, collagen hydrolysates (peptides): Current progress, challenges, and future perspectives. Food Chem..

[5] León-López A., Morales-Peñaloza A., Martínez-Juárez V.M., Vargas-Torres A., Zeugolis D.I., Aguirre-Alvarez G. (2019). Hydrolyzed Collagen-Sources and Applications. Molecules.

[6] Chen E.A., Lin Y.S. (2019). Using synthetic peptides and recombinant collagen to understand DDR-collagen interactions. Biochim. Biophys. Acta—Mol. Cell Res..

[7] He L., Lan W.T., Zhao Y.Q., Chen S.J., Liu S.L., Cen L.Y., Cao S., Dong L., Jin R.Y., Liu Y.W. (2020). Characterization of biocompatible pig skin collagen and application of collagen-based films for enzyme immobilization. Rsc Adv..

[8] Cao C.W., Wang H.L., Zhang J.Y., Kan H., Liu Y., Guo L., Tong H.Q., Wu Y.L., Ge C.R. (2023). Effects of Extraction Methods on the Characteristics, Physicochemical Properties and Sensory Quality of Collagen from Spent-Hens Bones. Foods.

[9] Jayaprakash S., Razeen Z.M.A., Kumar R.N., He J., Milky M.G., Renuka R., Sanskrithi M.V. (2024). Enriched characteristics of poultry collagen over other sources of collagen and its extraction methods: A review. Int. J. Biol. Macromol..

[10] Huang C.Y., Kuo J.M., Wu S.J., Tsai H.T. (2016). Isolation and characterization of fish scale collagen from tilapia (*Oreochromis* sp.) by a novel extrusion-hydro-extraction process. Food Chem..

[11] Vate N.K., Undeland I., Abdollahi M. (2022). Resource efficient collagen extraction from common starfish with the aid of high shear mechanical homogenization and ultrasound. Food Chem..

[12] Zhu N., Liu R., Xu M.H., Li Y. (2024). The Potential of Bioactive Fish Collagen Oligopeptides against Hydrogen Peroxide-Induced NIH/3T3 and HUVEC Damage: The Involvement of the Mitochondria. Nutrients.

[13] Chen L., Lv Y., Xu F., Zhong F. (2023). The effect of oral supplements containing collagen peptides rich in X-Hyp or X-Hyp-Gly compared with normal collagen hydrolysates on skin elasticity and collagen holes: A randomised double-blind clinical study. Food Funct..

[14] Yazaki M., Ito Y., Yamada M., Goulas S., Teramoto S., Nakaya M.A., Ohno S., Yamaguchi K. (2017). Oral Ingestion of Collagen Hydrolysate Leads to the Transportation of Highly Concentrated Gly-Pro-Hyp and Its Hydrolyzed Form of Pro-Hyp into the Bloodstream and Skin. J. Agric. Food Chem..

[15] Chang C.H., Ma Y.Z., Yang Y.J., Su Y.J., Gu L.P., Li J.H. (2024). Strategies to Improve Hydrolysis Efficiency of Fish Skin Collagen: Study on ACE Inhibitory Activity and Fibroblast Proliferation Activity. Foods.

[16] Tang C., Zhou K., Zhu Y., Zhang W., Xie Y., Wang Z., Zhou H., Yang T., Zhang Q., Xu B. (2022). Collagen and its derivatives: From structure and properties to their applications in food industry. Food Hydrocoll..

[17] Proksch E., Segger D., Degwert J., Schunck M., Zague V., Oesser S. (2014). Oral Supplementation of Specific Collagen Peptides Has Beneficial Effects on Human Skin Physiology: A Double-Blind, Placebo-Controlled Study. Ski. Pharmacol. Physiol..

[18] Albayati S.H., Nezhad N.G., Taki A.G., Abd Rahman R.N.Z.R. (2024). Efficient and feasible biocatalysts: Strategies for enzyme improvement. A review. Int. J. Biol. Macromol..

[19] Hu Z.Z., Sha X.M., Zhang L., Huang S., Tu Z.C. (2022). Effect of Grass Carp Scale Collagen Peptide FTGML on cAMP-PI3K/Akt and MAPK Signaling Pathways in B16F10 Melanoma Cells and Correlation between Anti-Melanin and Antioxidant Properties. Foods.

[20] Lee M., Kim D., Park S.-H., Jung J., Cho W., Yu A.R., Lee J. (2022). Fish Collagen Peptide (Naticol^®^) Protects the Skin from Dryness, Wrinkle Formation, and Melanogenesis Both In Vitroand In Vivo. Prev. Nutr. Food Sci..

[21] Taga Y., Tanaka K., Hattori S., Mizuno K. (2021). In-depth correlation analysis demonstrates that 4-hydroxyproline at the Yaa position of Gly-Xaa-Yaa repeats dominantly stabilizes collagen triple helix. Matrix Biol. Plus.

[22] Jiang S., Dong W.C., Zhang Z., Xu J., Li H.R., Zhang J.Y., Dai L., Wang S.P. (2022). A new iron supplement: The chelate of pig skin collagen peptide and Fe^2+^ can treat iron-deficiency anemia by modulating intestinal flora. Front. Nutr..

[23] Ni H., Liu C., Kong L.L., Zhai L.M., Chen J.P., Liu Q.P., Chen Z.D., Wu M.D., Chen J., Guo Y.Y. (2023). Preparation of injectable porcine skin-derived collagen and its application in delaying skin aging by promoting the adhesion and chemotaxis of skin fibroblasts. Int. J. Biol. Macromol..

[24] Hong G.P., Min S.G., Jo Y.J. (2019). Anti-Oxidative and Anti-Aging Activities of Porcine By-Product Collagen Hydrolysates Produced by Commercial Proteases: Effect of Hydrolysis and Ultrafiltration. Molecules.

[25] Zhou F., Li D., Hou Y., Cong Z., Li K., Gu X., Xiao G. (2024). Exploration of hypoglycemic peptides from porcine collagen based on network pharmacology and molecular docking. PLoS ONE.

[26] Lin X.L., Lu Y.J., Zhang T., Liang M., Cen Y.Y., Yuan E.D., Ren J.Y. (2019). Accuracy and Precision Comparison for Molecular Weight Distribution Assay of Fish Collagen Peptides: A Methodology Study Between Two Gel Permeation Chromatography Columns. Food Anal. Methods.

[27] Atma Y., Lioe H.N., Prangdimurti E., Seftiono H., Taufik M., Fitriani D., Mustopa A.Z. (2018). The hydroxyproline content of fish bone gelatin from Indonesian Pangasius catfish by enzymatic hydrolysis for producing the bioactive peptide. Asian J. Nat. Prod. Biochem..

[28] Qin D.D., Yang F.Y., Hu Z.M., Liu J.L., Wu Q., Luo Y., Yang L.F., Han S., Luo F.J. (2021). Peptide T8 isolated from yak milk residue ameliorates H_2_O_2_-induced oxidative stress through Nrf2 signaling pathway in HUVEC cells. Food Biosci..

[29] Chen Y.S., Liang Y.Y., He H., Sun M.Y., Huang P.T., Lin B.M., Zhan J.F., Cao Y., Miao J.Y. (2023). Optimisation process of walnut protein hydrolysed as an antioxidant candidate. J. Food Meas. Charact..

[30] Huina P., Yihan Y., Hongying D., Ting J., Hongyin Z., Yu Z., Tiequan C., Mingming Y., Shuai S. (2023). Structural properties of Kudzu protein enzymatic hydrolysate and its repair effect on HepG2 cells damaged by H_2_O_2_ oxidation. Food Funct..

[31] Wu W.M., He L.C., Liang Y.H., Yue L.L., Peng W.M., Jin G.F., Ma M.H. (2019). Preparation process optimization of pig bone collagen peptide-calcium chelate using response surface methodology and its structural characterization and stability analysis. Food Chem..

[32] Ueshima S., Yasumoto M., Kitagawa Y., Akazawa K., Takita T., Tanaka K., Hattori S., Mizutani K., Mikami B., Yasukawa K. (2023). Insights into the catalytic mechanism of Grimontia hollisae collagenase through structural and mutational analyses. FEBS Lett..

[33] Mokhtarnezhad V., Taheri A., Motamedzadegan A. (2021). Bioactive properties of marine fish skin gelatin hydrolysate: Optimisation using response surface methodology. Indian J. Fish..

[34] Awosika T., Aluko R.E. (2019). Enzymatic Pea Protein Hydrolysates Are Active Trypsin and Chymotrypsin Inhibitors. Foods.

[35] Ignat’eva N.Y., Danilov N.A., Averkiev S.V., Obrezkova M.V., Lunin V.V., Sobol E.N. (2007). Determination of hydroxyproline in tissues and the evaluation of the collagen content of the tissues. J. Anal. Chem..

[36] Zhang Z.J., Zhu H.W., Zheng Y.T., Zhang L.Y., Wang X.L., Luo Z., Tang J., Lin L., Du Z.Y., Dong C.Z. (2020). The effects and mechanism of collagen peptide and elastin peptide on skin aging induced by D-galactose combined with ultraviolet radiation. J. Photochem. Photobiol. B—Biol..

[37] Sontakke S.B., Jung J.H., Piao Z., Chung H.J. (2016). Orally Available Collagen Tripeptide: Enzymatic Stability, Intestinal Permeability, and Absorption of Gly-Pro-Hyp and Pro-Hyp. J. Agric. Food Chem..

[38] Zhang Y.H., Zhang Y.S., Liu X.M., Huang L.H., Chen Z.Y., Cheng J.R. (2017). Influence of hydrolysis behaviour and microfluidisation on the functionality and structural properties of collagen hydrolysates. Food Chem..

[39] Armin M., Marco G., JongBang E., Jesus S. (2022). Influence of Enzymatic Hydrolysis and Molecular Weight Fractionation on the Antioxidant and Lipase/α-Amylase Inhibitory Activities In Vitro of Watermelon Seed Protein Hydrolysates. Molecules.

[40] Yuswan M.H., Jalil N.H.A., Mohamad H., Keso S., Mohamad N.A., Yusoff T., Ismail N.F., Manaf Y.N.A., Hashim A.M., Desa M.N.M. (2021). Hydroxyproline determination for initial detection of halal-critical food ingredients (gelatin and collagen). Food Chem..

[41] Sun Y., Zhou C., Huang S., Jiang C. (2017). Selenium Polysaccharide SPMP-2a from Pleurotus geesteranus Alleviates H2O2-Induced Oxidative Damage in HaCaT Cells. BioMed Res. Int..

[42] Lee S., Rauch J., Kolch W. (2020). Targeting MAPK Signaling in Cancer: Mechanisms of Drug Resistance and Sensitivity. Int. J. Mol. Sci..

[43] Pillaiyar T., Manickam M., Namasivayam V. (2017). Skin whitening agents: Medicinal chemistry perspective of tyrosinase inhibitors. J. Enzym. Inhib. Med. Chem..

[44] Adzhubei A.A., Sternberg M.J.E., Makarov A.A. (2013). Polyproline-II Helix in Proteins: Structure and Function. J. Mol. Biol..

[45] Udenigwe C.C., Aluko R.E. (2012). Food protein-derived bioactive peptides: Production, processing, and potential health benefits. J. Food Sci..

[46] Hua Y.Y., Ma C.J., Wei T.T., Zhang L.F., Shen J. (2020). Collagen/Chitosan Complexes: Preparation, Antioxidant Activity, Tyrosinase Inhibition Activity, and Melanin Synthesis. Int. J. Mol. Sci..

[47] Huang P.T., Miao J.Y., Liao W.W., Huang C.S., Chen B.B., Li Y.K., Wang X.H., Yu Y., Liang X.T., Zhao H.S. (2023). Rapid screening of novel tyrosinase inhibitory peptides from a pearl shell meat hydrolysate by molecular docking and the anti-melanin mechanism. Food Funct..

[48] Asai T.T., Oikawa F., Yoshikawa K., Inoue N., Sato K. (2020). Food-Derived Collagen Peptides, Prolyl-Hydroxyproline (Pro-Hyp), and Hydroxyprolyl-Glycine (Hyp-Gly) Enhance Growth of Primary Cultured Mouse Skin Fibroblast Using Fetal Bovine Serum Free from Hydroxyprolyl Peptide. Int. J. Mol. Sci..

[49] Lin W., Yue Z., Zhiling Z., Fuping Z., Ruichang G. (2023). Food-derived collagen peptides: Safety, metabolism, and anti-skin-aging effects. Curr. Opin. Food Sci..

[50] Xue W.J., Liu X., Zhao W.Z., Yu Z.P. (2022). Identification and molecular mechanism of novel tyrosinase inhibitory peptides from collagen. J. Food Sci..

